# Heterogeneity of clinical and radiological findings of COVID-19

**DOI:** 10.1136/postgradmedj-2020-137901

**Published:** 2020-07-29

**Authors:** Giovanni D’Arena, Augusto La Penna, Antonino Crocamo, Francesca Sguazzo, Roberto Viceconti, Vincenzo Barlotti, Michele Gambardella

**Affiliations:** Hematology Service, S. Luca Hospital, Vallo Della Lucania (SA), Vallo Della Lucania, Italy; Radiology, S. Luca Hospital, Vallo Della Lucania (SA), Vallo Della Lucania, Italy; Infectious Disease, S. Luca Hospital, Vallo Della Lucania, Italy; Emergency, S. Luca Hospital, Vallo Della Lucania, Italy; Infectious Disease, S. Luca Hospital, Vallo Della Lucania, Italy; Infectious Disease, S. Luca Hospital, Vallo Della Lucania, Italy; Infectious Disease, S. Luca Hospital, Vallo Della Lucania, Italy

**Figure 1:**
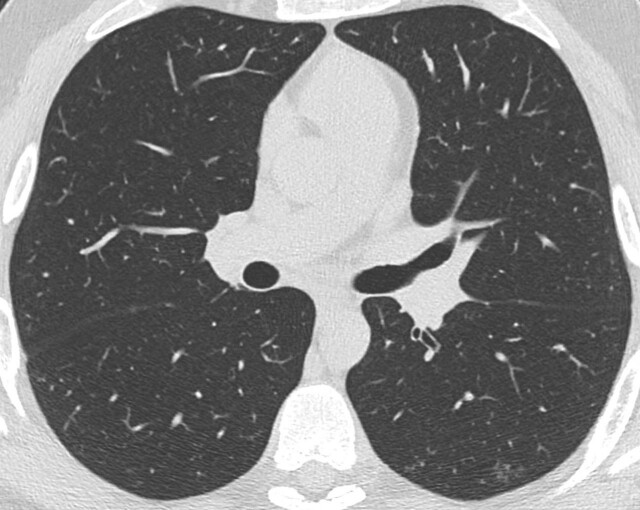
Thin slice (1 mm) lung CT of patients.

**Figure 2:**
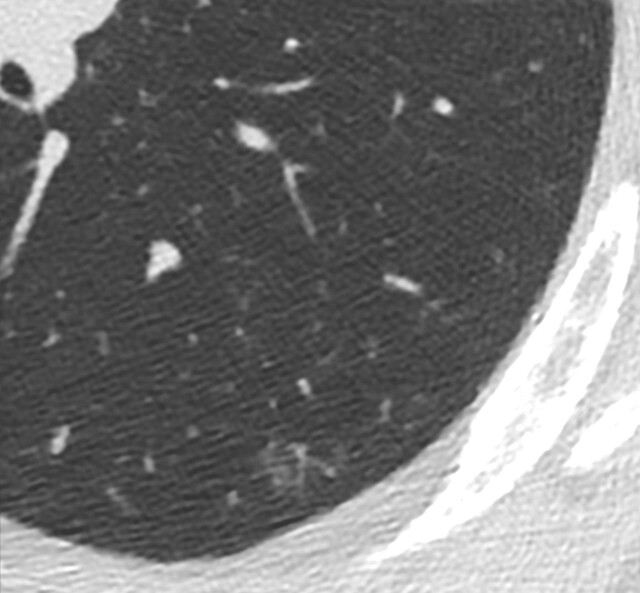
Singular intralobular GGO of a 31-year-old male patient with fever and cough. GGO, ground-glass opacity.

**Figure 3:**
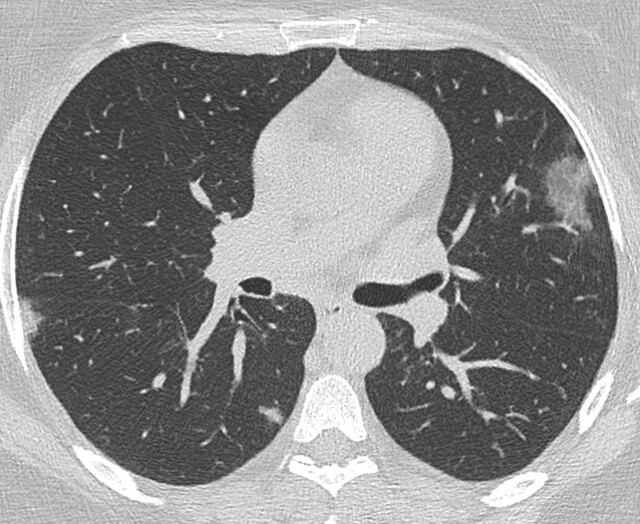
Bilateral patchy subpleural GGOs and small peripheral consolidation of a 56-year-old female with fever, cough and dyspnoea. GGO, ground-glass opacity.

**Figure 4:**
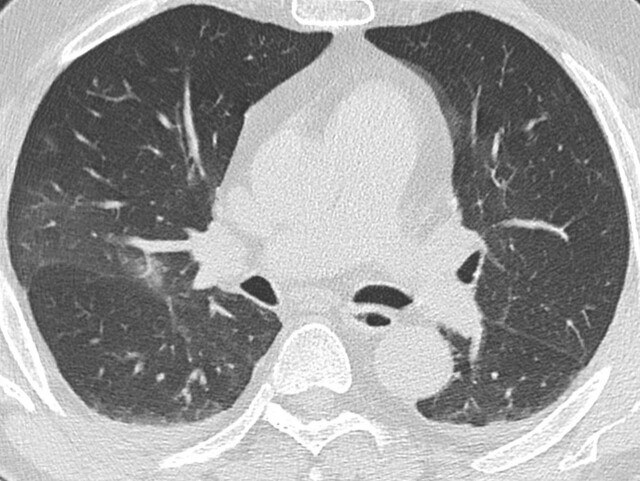
CT at presentation of a 62-year-old female with fever, cough and dyspnoea: perifissural GGOs in posterior segment of right upper lobe and small bilateral pleural effusion. GGO, ground-glass opacity.

**Figure 5:**
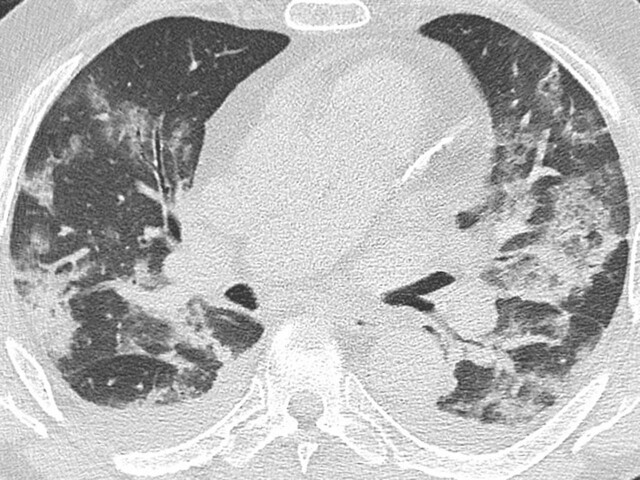
Follow-up at day 5: increased size and density of previous lesions, onset of new multiple bilateral GGOs and consolidations in both subpleural and central localisation, with interlobular septal thickening, and increased pleural effusion. GGO, ground-glass opacity

**Figure 6:**
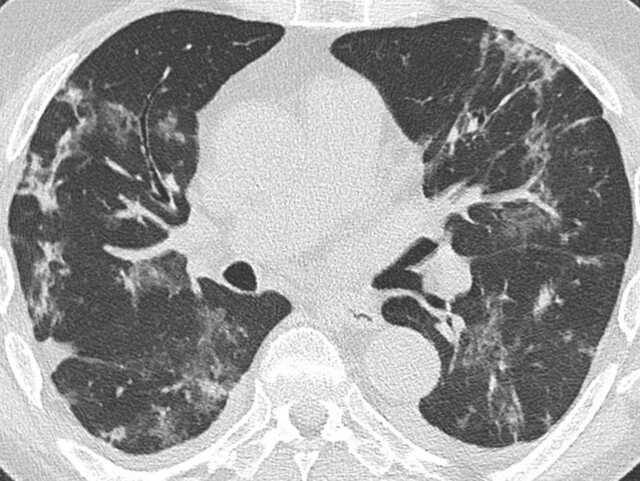
Follow-up at day 19: partial regression of the lesions with residual smaller GGOs, irregular parenchymal bands and interlobular septal thickening; reduction of bilateral pleural effusion with residual pleural fissure thickening and/or distortion. GGO, ground-glass opacity.

**Figure 7:**
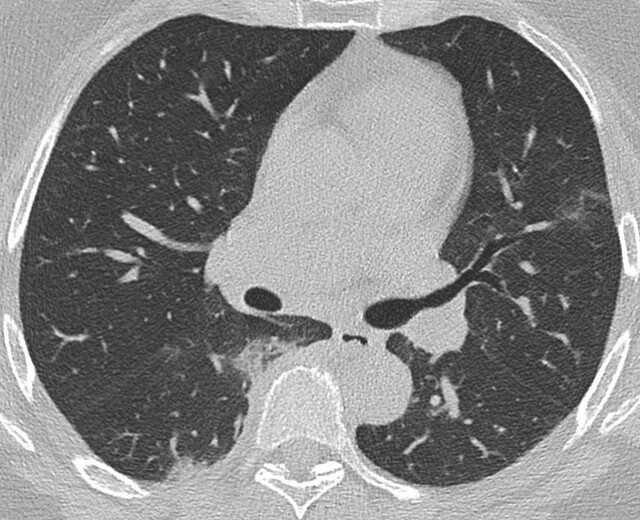
CT of a 38-year-old female with fever, cough, dyspnoea and anosmia at presentation: subpleural/peripheral GGOs. GGO, ground-glass opacity.

**Figure 8:**
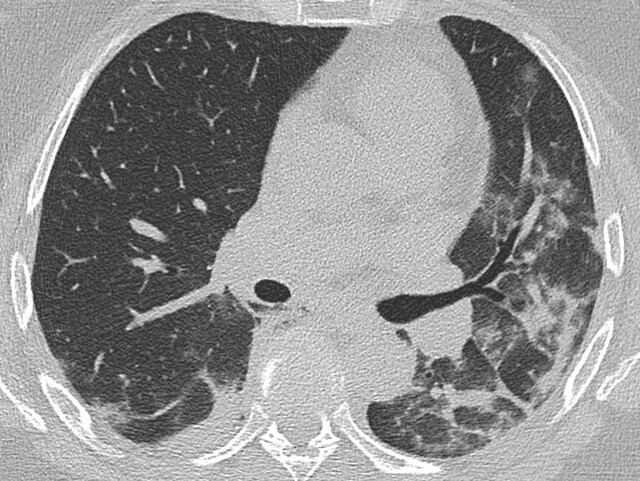
CT at day 9: increasing size and density of previous lesions, onset of new irregular consolidations with parenchymal bands and architectural distortion.

The pandemic COVID-19 caused by the 2019 novel coronavirus called severe acute respiratory syndrome coronavirus-2 displays a very heterogeneous clinical behaviour. The majority of patients (>85%) are asymptomatic or have mild symptoms, while few others show a very aggressive and life-threatening disease. Imaging spectrum of COVID-19 is very heterogenous as well: from normal picture in patients with mild symptoms such as fever and dry cough (figures 1 and 2) to pneumonia with multiple patchy, peripheral, bilateral areas of ground-glass opacity (GGO) and consolidation as in more severe illness (figures 3–8). A quick evolution of the disease is also seen. Involvement of both lungs seems to be the main imaging feature (75–100% of cases)^1  2^ usually with GGOs (77–91%)^1  3^ and consolidations (55–69%)^1  3^ in peripheral regions. Pleural effusions may occur in a minority of cases (4.1% of cases vs 39% in non-COVID-19 viral pneumonia)^1^; lymphadenopathy is rare, and pulmonary nodules and cavitation are not described.^1, 2, 3^ These imaging characteristics must be taken into account because they may help clinicians to better diagnose COVID-19 especially in an early phase^4^ and differentiate it from other viral cases of pneumonia (central distribution of lesions was observed in 80% vs 57% of cases in non-COVID-19 viral pneumonia in one study)^1^ or bacterial infections (usually with lobar or segmental consolidation).^5^
